# Providing Extrinsic Reward for Test Performance Undermines Long-Term Memory Acquisition

**DOI:** 10.3389/fpsyg.2016.00079

**Published:** 2016-02-01

**Authors:** Christof Kuhbandner, Alp Aslan, Kathrin Emmerdinger, Kou Murayama

**Affiliations:** ^1^Department of Psychology, University of RegensburgRegensburg, Germany; ^2^Department of Psychology, Martin Luther University of Halle-WittenbergHalle, Germany; ^3^Department of Psychology, University of ReadingReading, UK

**Keywords:** testing effect, motivation, high-stakes testing, long-term memory, monetary reward

## Abstract

Based on numerous studies showing that testing studied material can improve long-term retention more than restudying the same material, it is often suggested that the number of tests in education should be increased to enhance knowledge acquisition. However, testing in real-life educational settings often entails a high degree of extrinsic motivation of learners due to the common practice of placing important consequences on the outcome of a test. Such an effect on the motivation of learners may undermine the beneficial effects of testing on long-term memory because it has been shown that extrinsic motivation can reduce the quality of learning. To examine this issue, participants learned foreign language vocabulary words, followed by an immediate test in which one-third of the words were tested and one-third restudied. To manipulate extrinsic motivation during immediate testing, participants received either monetary reward contingent on test performance or no reward. After 1 week, memory for all words was tested. In the immediate test, reward reduced correct recall and increased commission errors, indicating that reward reduced the number of items that can benefit from successful retrieval. The results in the delayed test revealed that reward additionally reduced the gain received from successful retrieval because memory for initially successfully retrieved words was lower in the reward condition. However, testing was still more effective than restudying under reward conditions because reward undermined long-term memory for concurrently restudied material as well. These findings indicate that providing performance–contingent reward in a test can undermine long-term knowledge acquisition.

## Introduction

A central question of both experimental research and educational practice is how learning and retention can be promoted. A very powerful technique to improve long-term memory seems to be retrieving previously learned materials while taking a test (e.g., Gates, 1917; see Roediger and Butler, 2011 for a review). Several recent studies have renewed interest in this phenomenon by demonstrating that retrieving materials in a test promotes even better long-term retention than restudying the same materials (e.g., Roediger and Karpicke, 2006a; Karpicke and Roediger, 2008), a phenomenon called “test-enhanced learning”. In view of such findings, it has been recommended that the number of tests in education should be increased as frequent testing may boost students’ achievement (Roediger and Karpicke, 2006b).

However, in real-life educational settings, test-taking may have additional effects on the emotions and motivations of learners, factors that have been largely neglected in previous research on the effect of testing. This neglect is particularly interesting because there is reason to assume that such effects may undermine the effectiveness of testing in enhancing long-term memory. For instance, regarding emotions, if a test induces a high degree of performance-related anxiety, the reduction in cognitive resources due to distraction by task-irrelevant emotion-induced thoughts (e.g., Ellis and Ashbrook, 1988) may impair cognitive processes underlying the enhancement of long-term learning. Indeed, this is supported by a recent study showing that performance pressure-induced test anxiety can attenuate the beneficial effects of a test on long-term memory (Hinze and Rapp, 2014).

At the motivational level, a typical effect of testing in real-life educational settings is that the motivation of learners is shifted toward an extrinsically motivated state due to the common practice of placing important consequences on the outcome of a test (for a review, see Harlen and Crick, 2003). Basically, whereas intrinsically motivated behaviors are engaged for their own sake, extrinsically motivated behaviors are driven by the prospect of instrumental gains and losses (e.g., Deci, 1971; Ryan and Deci, 2000; Cerasoli et al., 2014). Critically, with regard to learning, numerous studies have shown that the quality of learning varies as a function of the motivational state of learners. Whereas intrinsically motivated learners show a more elaborative learning style characterized by more active and effortful learning that persists beyond the point of being rewarded or punished, extrinsically motivated learners show a more superficial learning style characterized by more passive and less effortful learning that vanishes beyond the point of being rewarded or punished (e.g., Benware and Deci, 1984; Simons et al., 2004; Vansteenkiste et al., 2004; Lepper et al., 2005). Accordingly, it may be that when the taking of a test leads to a high degree of extrinsic motivation, the detrimental effects of extrinsic motivation on learning may undermine the memory-enhancing effect of retrieving learned material in a test.

Basically, there are two possibilities why a test that induces a high degree of extrinsic motivation may undermine the effectiveness of testing in enhancing long-term knowledge acquisition. First, by providing gains contingent on performance, such a test induces a strong desire to perform as well as possible. Such a desire may impair the quality of retrieval of actually stored knowledge. On the one hand, the rate of successfully retrieved information may be decreased because it has been shown that people often perform below actual abilities when trying to perform as well as possible, an observation that is commonly attributed to the experience of performance pressure. Such performance pressure often leads to the occupation of attention by task-irrelevant thoughts, such as ruminations about one’s performance and its consequences (e.g., Baumeister, 1984; DeCaro et al., 2011). On the other hand, the rate of erroneously retrieved information (i.e., commission errors) may be increased because people may try to maximize their gains by guessing (e.g., Legault and Inzlicht, 2013). An increased rate of commission errors in a test may be problematic for long-term learning because learners may store the erroneously retrieved information in long-term memory, with the detrimental consequence that they may acquire erroneous knowledge (e.g., Roediger and Karpicke, 2006b). These detrimental effects of extrinsic reward on the retrieval of learned material in a test may decrease the benefits gained from testing for long-term memory.

Second, a test that induces extrinsic motivation may even reduce the benefit received from successful retrieval. Most theoretical accounts proposed to explain the high effectiveness of testing assume that retrieval of information from memory represents a new learning event (i.e., reconsolidation; e.g., Dudai, 2004) that allows storing the retrieved information more elaborately and deeply (e.g., Finn and Roediger, 2011; Finn et al., 2012; see Roediger and Butler, 2011, for a review). However, if a test is taken in an extrinsically motivated state, such reconsolidation processes may be weakened due to the more passive and less persistent learning brought about by extrinsic motivation (e.g., Benware and Deci, 1984; Grolnick and Ryan, 1987; Vansteenkiste et al., 2004).

The aim of the present study was to examine the effect of extrinsic motivation on the long-term memory effects of testing. To examine the issue, we employed a standard testing-effect paradigm and manipulated the degree of extrinsic motivation during immediate testing. Participants first studied Swahili–German vocabulary pairs (e.g., Mashua–Boat) without mentioning that they may be rewarded for their later test performance. In a subsequent immediate memory test, one-third of the vocabulary pairs were tested, one-third were presented for restudy, and the remaining third did not appear in the test and served as control pairs. In order to manipulate the degree of extrinsic motivation during immediate testing, participants received either performance-contingent monetary reward for test performance (high extrinsic-motivation condition), or not (low extrinsic-motivation condition; e.g., Murayama et al., 2010). To control for potential confounding effects of receiving money on post-learning consolidation processes (e.g., Nielson and Bryant, 2005; Murayama and Kitagami, 2014), participants in the low extrinsic-motivation condition received money as well. However, other than in the high extrinsic-motivation condition, this money was not performance-contingent and not framed as a reward. Instead, participants took part in a lottery, and they were told that they can earn some additional remuneration for participating in the experiment. Then, after a delay of 1 week, memory for all initially studied vocabulary pairs was tested.

In the immediate test, we expected to replicate the detrimental effects of providing extrinsic reward contingent on test performance on the quality of retrieval of learned knowledge (e.g., Baumeister, 1984; DeCaro et al., 2011; Legault and Inzlicht, 2013); that is, we expected that the rate of successfully retrieved information would be decreased and the rate of commission errors would be increased. If so, then memory in the delayed test for initially tested vocabulary pairs should be impaired in the high compared to the low extrinsic-motivation condition as well because less vocabulary pairs can benefit from being initially successfully retrieved. If extrinsic motivation additionally undermines the benefit received from successful retrieval, then memory for initially successfully retrieved vocabulary pairs in the delayed test should be reduced in the high compared to the low extrinsic-motivation condition as well. Regarding the effect of extrinsic motivation on restudied items, it may be that concurrently restudied items suffer less from extrinsic motivation because the problem of retrieval impairment is circumvented when all information is presented again for restudy. If so, the advantage of testing over restudying should be decreased in the high compared to the low extrinsic-motivation conditions. However, extrinsic motivation may lead to a less effortful restudying of concurrently presented but not rewarded information. If so, despite the detrimental effect of extrinsic motivation on the effects of testing, the advantage of testing over restudying should be similar between the high and low extrinsic-motivation conditions.

## Materials and Methods

### Participants

Sixty undergraduate students (49 females; *M* = 22.9 and *SD* = 4.3 years) participated in the experiment for course credit. Participants were tested in small groups of up to five individuals. One of the original participants was replaced (in the low extrinsic-motivation condition) because he did not recall a single item in the immediate test. Including this participant did not change the significance of any of our results. The study was conducted in accordance with the Helsinki declaration and the University Research Ethics Standards.

### Materials

The study list consisted of 30 Swahili–German vocabulary pairs drawn from Karpicke and Roediger (2008).

### Design and Procedure

Half of the participants were randomly assigned to a low extrinsic-motivation condition, and the other half to a high extrinsic-motivation condition. In each condition, the experiment consisted of three main phases: a study phase, a (combined) test/restudy phase, and a 1-week delayed final test phase. In the study phase, participants were presented 30 vocabulary pairs (e.g., *Mashua* – Boat, *Bustani* – Garden, *Farasi* – Horse) in randomized order. Stimuli were delivered via a projector at a 7-s rate with an interstimulus interval of 1 s. Participants were asked to read the vocabulary pairs silently and memorize them for a later cued-recall test (e.g., *Mashua* – ?). It was not mentioned that they may be rewarded for their later test performance. Following presentation of the last pair, the whole list was presented a second time. In the subsequent test/restudy phase, participants were tested on one-third of the vocabulary pairs (without any feedback) by providing the Swahili words as retrieval cues for the German words (tested vocabulary pairs; e.g., *Mashua* – ?), while another third of the pairs were re-presented to the participants for restudying (restudied vocabulary pairs; e.g., *Bustani* – Garden); the remaining third of vocabulary pairs did not appear in this phase and served as a baseline for the benefits gained from testing and restudying (control vocabulary pairs). The stimuli were delivered via a projector and participants were instructed to write down both of the two words of a vocabulary pair within 10 s, both for the test and restudy pairs. The order of the 10 test and 10 restudy trials was randomized, and the assignment of the vocabulary pairs to the three learning conditions was counterbalanced. Directly before the test/restudy phase, participants in the high extrinsic-motivation condition were encouraged to perform as well as possible on the test trials because they were told they would be paid 1 Euro for each correctly recalled German word. No such instruction was given in the low extrinsic-motivation condition. In order to control for potential confounding effects of receiving money on post-learning consolidation processes (e.g., Nielson and Bryant, 2005; Murayama and Kitagami, 2014), participants in the low extrinsic-motivation condition also received money. In contrast to the high extrinsic-motivation condition, however, this remuneration was not related to their recall performance and was not framed as ‘reward’. Instead, participants were told that they can take part in a lottery where they could earn some additional remuneration for participating in the experiment (additionally to the course credit they received for participation). The sums of money that individual participants won in the lottery were adjusted so that each participant in the low extrinsic-motivation condition was monetarily yoked to a participant in the high extrinsic-motivation condition so that, across participants, the mean amount of received money was equal in the two conditions. After immediate testing, all participants were asked to return to the laboratory 1 week later for a delayed cued-recall test covering all initially studied vocabulary pairs, and they were informed that the delayed memory test would be unpaid. Upon arrival in the laboratory 1 week later, participants were given a sheet of paper including the 30 Swahili words in randomized order, and were asked to recall and write down the corresponding German words. There was no time restriction in this test. After completion of the delayed memory test, participants were thanked and they received their money.

## Results

### Immediate Test

**FIGURE 1:**
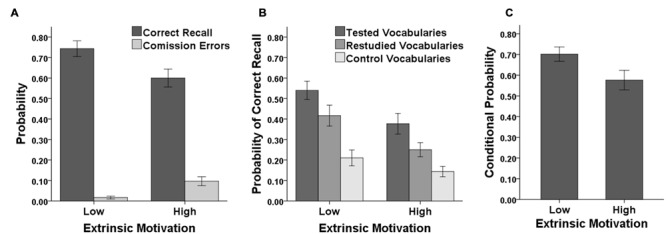
**Results of the Experiment. (A)** Probability of correct recall and commission errors in the immediate memory test as a function of extrinsic motivation (low and high). **(B)** Probability of correct recall in the delayed long-term memory test as a function of item type (tested, restudied, and control) and extrinsic motivation (low and high). **(C)** Conditional probability of correct recall in the delayed long-term memory test given successful recall in the immediate memory test as a function of extrinsic motivation (low and high). Error bars represent standard errors of the means.

Memory performance in the immediate test as a function of motivational condition is shown in **Figure 1A**. Probability of correct recall was lower in the high than the low extrinsic-motivation condition (high: *M* = 0.60, *SD* = 0.24 vs. low: *M* = 0.74, *SD* = 0.21), *t*(58) = -2.47, *p* = 0.017, *d* = 0.64, whereas the probability of commission errors (intra-list intrusions) was higher in the high than the low extrinsic-motivation condition (high: *M* = 0.10, *SD* = 0.12 vs. low: *M* = 0.02, *SD* = 0.04), *t*(58) = 3.51, *p* < 0.001, *d* = 0.91.

### Delayed Test

**Figure 1B** shows memory performance in the delayed test for initially tested, restudied, and control vocabulary pairs as a function of motivational condition. A 2 × 3 ANOVA with the within-participants factor of vocabulary type (tested, restudied, and control) and the between-participants factor of extrinsic motivation (high and low) revealed a significant main effect of vocabulary type, *F*(2,116) = 47.48, *p* < 0.001, $\eta_{p}^{2}$ = 0.450, reflecting the fact that correct recall was higher for restudied than for control vocabulary pairs (*M* = 0.33, *SD* = 0.25 vs. *M* = 0.18, *SD* = 0.18), *t*(59) = 5.60, *p* < 0.001, *d* = 0.72, and even higher for tested than for restudied vocabulary pairs (*M* = 0.46, *SD* = 0.27), *t*(58) = 4.15, *p* < 0.001, *d* = 0.48. The main effect of extrinsic motivation was also significant, *F*(1,58) = 7.40, *p* = 0.009, $\eta_{p}^{2}$ = 0.113, reflecting the fact that, collapsed across the three vocabulary types, correct recall was lower in the high than the low extrinsic-motivation condition (high: *M* = 0.26, *SD* = 0.17 vs. low: *M* = 0.39, *SD* = 0.21). The interaction between vocabulary type and extrinsic motivation was not significant, *F*(2,116) = 1.92, *p* = 0.151, $\eta_{p}^{2}$ = 0.032. Simple main effect analyses showed that correct recall for tested vocabulary pairs was lower in the high than the low extrinsic-motivation condition (high: *M* = 0.38, *SD* = 0.28 vs. low: *M* = 0.54, *SD* = 0.24), *t*(58) = -2.43, *p* = 0.018, *d* = 0.63, indicating that initial reward reduced memory performance for tested contents. Correct recall for restudied vocabulary pairs was lower in the high than the low extrinsic-motivation condition as well (high: *M* = 0.25, *SD* = 0.19 vs. low: *M* = 0.42, *SD* = 0.28), *t*(58) = -2.71, *p* = 0.009, *d* = 0.70, indicating that the detrimental effects of reward transferred to restudied items. Correct recall for control vocabulary pairs did not significantly differ between motivational conditions (high: *M* = 0.14, *SD* = 0.14 vs. low: *M* = 0.21, *SD* = 0.21), *t*(58) = -1.45, *p* = 0.153, *d* = 0.37.

A 2 × 3 ANOVA with the within-participants factor of vocabulary type (tested, restudied, and control) and the between-participants factor of extrinsic motivation (high and low) on the probability of commission errors (intra-list intrusions) revealed neither a main effect of vocabulary type nor a main effect of extrinsic motivation, *F*s < 2.48 and *p*s > 0.121, but a significant interaction, *F*(1,58) = 3.33, *p* = 0.039, $\eta_{p}^{2}$ = 0.054. Simple main effect analyses showed that whereas commission errors did not differ between motivational conditions for restudied (high: *M* = 0.03, *SD* = 0.07 vs. low: *M* = 0.02, *SD* = 0.05) and control vocabulary pairs (high: *M* = 0.03, *SD* = 0.05 vs. low: M = 0.043, *SD* = 0.06), *t*s < 0.70 and *p*s > 0.490, for tested vocabulary pairs commission errors were observed more often in the high than the low extrinsic-motivation condition (high: *M* = 0.07, *SD* = 0.09 vs. low: *M* = 0.020, *SD* = 0.05), *t*(58) = 2.54, *p* = 0.014, *d* = 0.66.

Finally, we examined the effect of reward on memory for vocabulary pairs which were initially successfully retrieved. To control for potential item-selection artifacts (i.e., artifacts due to unbalanced distribution of vocabulary pairs across conditions because of differential recall in the immediate memory test), we determined for each vocabulary pair the conditional probability of correct recall in the delayed test given successful recall in the immediate test, collapsing data across participants. As shown in **Figure 1C**, conditional probability of correct recall was lower in the high than the low extrinsic-motivation condition (high: *M* = 0.58, *SD* = 0.26 vs. low: *M* = 0.70, *SD* = 0.19), *t*(29) = 2.70, *p* = 0.011, *d* = 0.49, indicating that even initially successfully retrieved vocabulary pairs benefited less from testing when extrinsic motivation was high.

## Discussion

Previous research has shown that retrieving previously learned contents in a test can improve long-term memory for tested contents, suggesting that the number of tests in education should be increased to enhance knowledge acquisition (see Roediger and Karpicke, 2006b, for a review). The present study demonstrates, however, that the effect of retrieval is undermined when a test entails a high degree of extrinsic motivation due to the provision of gains contingent on test performance. Compared to a no-reward condition, rewarding participants with money depending on performance in the immediate test decreased correct recall and increased commission errors for tested contents after 1 week. Thus, given that the placing of important consequences on the outcome of a test is common practice in educational settings, the consequences of testing in education on the acquisition of knowledge for later life and work may be less encouraging than previously believed.

More detailed analyses showed that the detrimental effects of reward were attributable to two factors. First, the provision of monetary reward contingent on performance reduced correct recall and increased commission errors in the immediate test, a pattern that typically occurs in situations where people try to perform as well as possible to maximize promised extrinsic gains (e.g., Baumeister, 1984; DeCaro et al., 2011; Legault and Inzlicht, 2013). Such an effect of reward on immediate test performance seems to have two negative consequences for later long-term memory. On the one hand, by decreasing the amount of information that is successfully retrieved, reward seems to reduce the amount of stored information that can benefit from retrieval (e.g., Bjork and Bjork, 1992; Kornell et al., 2011). On the other hand, by increasing the amount of information that is erroneously retrieved, reward seems to increase the degree of information that is erroneously reconsolidated. This is reflected by the fact that commission errors in the delayed test were increased in the reward condition for vocabulary pairs that were part of the immediate memory test, but not for restudied and control vocabulary pairs that were not actively retrieved during immediate testing.

Second, even for vocabulary pairs that were initially successfully retrieved, long-term memory was reduced when reward was initially provided. Thus, extrinsic motivation seems to undermine even the benefit gained from successfully retrieving stored information in a test. Such an effect is consistent with findings showing that the quality of learning differs depending on motivational state. Compared to intrinsic motivation, extrinsically motivated learners show a less elaborative learning style characterized by more passive and less effortful learning that vanishes beyond the point of being rewarded or punished (e.g., Benware and Deci, 1984; Grolnick and Ryan, 1987; Vansteenkiste et al., 2004; Lepper et al., 2005). Such detrimental effects of extrinsic motivation may reduce the memory-enhancing effects of testing by reducing the quality of learning evoked by retrieval.

The present results further show that high extrinsic motivation can even have detrimental effects on long-term memory for material that is restudied. Compared to the no-reward condition, memory in the delayed test for vocabulary pairs that were initially restudied was reduced in the reward condition as well. As participants were forced to write down each of the to-be-restudied vocabulary pairs during restudy, such a finding cannot easily be explained by the simple assumption that rewarding participants only for some vocabulary pairs led them to abandon processing of not rewarded vocabulary pairs. However, in such a situation, the induced extrinsic motivation seems to bring about a less effortful restudying of not rewarded information.

In the present study, we examined the effect of providing performance-contingent reward in an immediate memory test on performance in a delayed long-term memory test where no reward was provided. This situation mimics the typical educational scenario in which the objective is to provide learners with knowledge to prepare them for later life and work, where knowledge retrieval is not necessarily driven by extrinsic forces. Doing so, we found that providing extrinsic reward for test performance can undermine long-term knowledge acquisition of the assessed contents. The situation may be different however, when extrinsic motivation is increased during immediate test-taking because learners are aware that they are preparing for a delayed test for which they will be rewarded based on their performance. In such a situation, additional motivational factors may play an important role during immediate test taking, such as the motivation to learn the material as well as possible for the delayed test (see Hidi and Harackiewicz, 2000, for a review). As a result, this may attenuate the detrimental effects of extrinsic motivation on the quality of learning. Indeed, this assumption is supported by a recent study, showing that the prospect of receiving monetary reward for performance in the delayed test seems not to reduce the beneficial effects testing (Kang and Pashler, 2014). Still, it seems possible that long-term knowledge acquisition beyond the delayed test for which reward was provided suffers from the increase in extrinsic motivation, which is an issue that should be explored in future research.

The present study also raises several questions that should be addressed in future research. First, our sample consisted mainly of female undergraduate students. Thus, future research should examine whether the results of the present study generalize across gender and different levels of education. Second, in order to be able to relate our results to prior findings, the study material consisted of foreign language word pairs that have been frequently used in research on the effects of testing (e.g., Karpicke and Roediger, 2008; Kang and Pashler, 2014). Thus, future research should examine whether the results of the present study generalize across other types of study materials such as text passages or general knowledge facts. Third, because all participants participated for course credit, learning in the condition where no reward was provided for test performance was not entirely intrinsically motivated. Our prediction would be that the detrimental effect of reward may be even more noticeable when compared to a condition where participants participate without receiving any reward because their motivational state is then shifted even more strongly toward an intrinsically motivated state, a prediction that deserves future research.

Finally, the present results may have important implications for applied settings, such as educational practice. Based on the finding that test taking can enhance later memory, it has been argued that increasing the number of tests in education is a promising technique to boost educational achievement (e.g., Roediger and Karpicke, 2006b). Our findings demonstrate that the effectiveness of testing in improving long-term knowledge acquisition is reduced when a test leads to a high degree of extrinsic motivation due to the provision of performance-contingent reward. Therefore, the common practice to implement tests as high-stakes assessments which have to be passed in order to reach important benefits may counteract the beneficial effects of testing on the acquisition of knowledge in long-term memory. One possibility to at least partly overcome the detrimental effects of reward may be to provide corrective feedback as this would reduce the problem of retrieval impairment due to the desire to perform as well as possible. However, as extrinsic motivation even seems to decrease the memory strength gained from successful retrieval and from restudying concurrently presented contents, tests that lead to high extrinsic motivation may still be less effective than tests that do not increase extrinsic motivation. Thus, if possible, educators would be well advised to implement tests as low-stakes assessments, in order to maximize the effectiveness of testing for long-term knowledge acquisition.

## Author Contributions

CK developed the study concept. All authors contributed to the study design and all authors analyzed and interpreted the data. CK prepared the draft manuscript, and AA, KE, and KM provided critical revisions. All authors approved the final version of the manuscript for submission.

## Conflict of Interest Statement

The authors declare that the research was conducted in the absence of any commercial or financial relationships that could be construed as a potential conflict of interest.

The reviewer Michael S. Dempsey and handling Editor declared their shared affiliation, and the handling Editor states that the process nevertheless met the standards of a fair and objective review.
